# Assessment of Unilateral Spatial Neglect Using a Free Mobile Application for Italian Clinicians

**DOI:** 10.3389/fpsyg.2018.02241

**Published:** 2018-11-22

**Authors:** Pietro Cipresso, Elisa Pedroli, Silvia Serino, Michelle Semonella, Cosimo Tuena, Desirée Colombo, Federica Pallavicini, Giuseppe Riva

**Affiliations:** ^1^Applied Technology for Neuro-Psychology Lab, Istituto Auxologico Italiano, Milan, Italy; ^2^Department of Psychology, Università Cattolica del Sacro Cuore, Milan, Italy; ^3^Department of Basic Psychology, Clinic and Psychobiology, Universitat Jaume I, Castellón de la Plana, Spain; ^4^“Riccardo Massa” Department of Human Sciences for Education, University of Milano-Bicocca, Milan, Italy

**Keywords:** neglect, psychometrics, computational psychometrics, ecological assessment, mobile virtual reality, mHealth, pervasive computing, mental health

## Abstract

**Background:** Unilateral Spatial Neglect (USN) is traditionally assessed with paper-and-pencil tests or computer-based tests. Thanks to the wide-spreading of mobile devices, and the extensive capabilities that they have in dealing complex elements, it is possible to provide clinicians with tools for cognitive assessment. Contemporary 3D engine is, in general generally, able to deploy complex 3D environments for iOS, Android and Windows mobile, i.e., most of the mobile phone and tablet operative systems.

**Results:** This brand-new scenario and pressing requests from professionals, pushed us to build an application for the assessment of USN. Our first attempt was to replicate the classic cognitive tests, traditionally used at this purpose. Ecological assessment is difficult in real scenarios so we implemented virtual environments to assess patients’ abilities in realistic situations. At the moment, the application is available only for iPad and iPhone for free, from the Apple Store, under the name of “Neglect App.” The App contains traditional tests (e.g., barrage with and without distractors) and ecological tests (e.g., to distribute the tea in a table to close people). Scoring of each test is available to the clinicians through a database with the executed ecological tasks, that are stored locally.

**Conclusion:** In conclusion, Neglect App is an advanced mobile platform for the assessment of Neglect.

## Introduction

The Unilateral Spatial Neglect (USN) or Neglect manifests in about 2/3 of patients during the acute phase following a stroke. Stroke is an occurrence of cerebral vascular disease resulting in acute disruption of the focal or generalized brain function. Every year, there are approximately 500,000 stroke patients in Europe. This is the third leading cause of death in Western countries after cardiovascular diseases and malignancies (Sudlow and Warlow, 1997; Pendlebury et al., 2009; Roger et al., 2012; Go et al., 2014; Mozaffarian et al., 2015). A stroke is a catastrophic and often unexpected event with a wide range of physical and psychological consequences in the long term for both patients and their families.

The long-term effects of stroke depend on the type, severity, and location of the occlusion: it is important to identify as soon as possible which part of the brain and how severely it has been affected. In general, two basic categories of impairments or disabilities can be identified: cognitive disability, which includes memory problems, difficulty in executive functions and aphasia, and motor disabilities, which includes the inability to walk and problems with coordination and balance (ataxia), mobility difficulties with arms, hemiparesis or hemiplegia, spasticity and contractures.

In particular USN can be defined as a disorder because the patient has difficulties to explore, pay attention, perceive, and act within the space opposite the region of the brain lesion. Often, there is also a difficulty in elaborating mental images in the opposite side of the damaged one. It is important to underline that the problems shown by patients are not caused by primary sensory or motor deficit, although they are often associated with hemiplegia and hemianopia (Ducros, 2012; Vocat et al., 2013; Heilman, 2014; Saj et al., 2014). These problems occur mainly following a damage to the right brain hemisphere, but there are patients in which the syndrome arose after a left-sided lesion; right neglect is considered less severe and less enduring (Stone et al., 1991; Halligan and Robertson, 2014). Regardless the side of the lesion, this disorder can be caused by the damage of several areas; the most typical one is the parietal lobe, specifically the inferior parietal lobule, followed by the frontal lobe and other sub-cortical structures such as the thalamus and the basal ganglia (Moretti et al., 2012; Saj et al., 2012; Antal et al., 2014).

In the acute phase and in the more severe form, the patient appears with the head and the gaze turned to the ipsilesional side, insensitive to any stimulation coming from the contralesional side. Over time, symptoms may ease, although more and more studies are showing that the disorder can last even for years (Kerkhoff and Schenk, 2012).

The neglect can be accompanied by several phenomena:

– Anosognosia: the unawareness of his/her own disability does not allow the patient to formulate strategies to compensate the lack of exploration in the left space (something that occurs when patients are affected by hemiplegia or hemianopsia; Bisiach et al., 1986; Pia et al., 2004).
– Anosodiaphoria: indifference or inadequate emotional response showing as the awareness of the disease increases (Barré et al., 1923; Migliaccio et al., 2014; Gasquoine, 2015).
– Extinction to double stimulation: the patient, who is able to identify a single stimulus presented on the contralesional side, cannot recognize it if presented together with an ipsilesional one (Vossel et al., 2011; de Haan et al., 2012; Heidler-Gary et al., 2013).
– Allochiria: the patient transports a stimulus to the neglected side to the ipsilesional one. For example, in case of left-sided neglect, if touched on his/her left leg the patient mentions to have being touched on the right one (Treccani et al., 2012; Antoniello and Gottesman, 2013; Marshall et al., 2013; Bartolomeo, 2014).

The standard neuropsychological tests for the analysis of extra personal neglect can be divided into:

– *cancellation test*: tasks requiring that the patient deletes certain elements within a spreadsheet, alone or mixed with distracters (Albert, 1973; Diller et al., 1974; Halligan and Marshall, 1989).
– *reading test*: both words and phrases (Pizzamiglio et al., 1989a,b) evaluate what is called neglect dyslexia.
– *bisection of lines*: the patient is required to mark the half of lines of different lengths and place in different ways in the space.
– *copy of drawings*: the patient has to copy a complex figure such as a daisy.

What may be of great help to clinical placement is real exploration of space such as the room, where the patient is hospitalized to or the one where tests are conducted to have more complete picture of the patient’s spatial abilities. Unfortunately, it is difficult to make these tests in a clinical setting because of the higher requested time and human resources. Finally, it is difficult to standardize these tests due to the heterogeneity of the experimental situations.

The purpose of this App was to include the described tests for a portable and electronic use, including also an automated score recording, that can also help in simplifying the difficult process of neuropsychological assessment. In one hand several tests have been included, as it is shown in the following sections. On the other hand, we made the effort of including new paradigms and tests that are difficult to be made in paper and pencil mode. In particular, navigation tasks and ecological tests represent our effort integrating current paradigms for neuropsychological assessment. At the moment, a plaint of possible features and indexes are probably still missing, however, the App represent the first effort ever in integrating many tests and tasks in a mobile application. This could be the first step toward future integrations.

## Implementation

Neglect App is the first application for mobile devices which makes use of the huge potential of virtual environments for the assessment of the USN for which an evaluation as effective and prompt as possible are crucial (Pallavicini et al., 2015; Pedroli et al., 2015a,b, 2016).

During the process of the design of the App, we also exploited the potential of 3D interactive applications for preventing and/or improving cognitive impairments related to USN, on the basis of a series of advantages amply documented by scientific literature:

### Neuroplasticity

Neuroplasticity: the App permits to use scenarios specifically designed following principles that regulate and facilitate neuroplasticity (the neurobiological process basis of recovery of cognitive and motor functions), such as exercise intensity, exercise frequency, “enriched stimulation” (Cheung et al., 2014; Ekman et al., 2018).

### Personalized Training

Personalized training: the App is based on highly automated functioning mechanisms that requires a minimal contribution by the clinical therapists, who have the possibility to customize the intensity and the difficulty of the training based on the specific needs of the patients; Engaging tasks: in the App, the content of training exercises are based on defining some tasks to re-train specific abilities (for example, increasing complexity time by time), and in the same time integrating in the scenario some recreational elements to maintain a high level of engagement and compliance of the older participant. Specifically, ecological simulations can be particularly engaging by supporting a process known as “transformation of flow,” defined as a person’s ability to exploit an optimal (flow) experience to identify and use new and unexpected psychological resources as sources of involvement (Riva et al., 2006; Pedroli et al., 2018). Also, presence is a key point of the engagement in the use of technology. Presence is usually defined as the “sense of being there” or the “feeling of being in a world that exists outside the self.” The ability to interact actively with the environment greatly improves the possibility of experiencing presence (Riva et al., 2007; Villani et al., 2012b).

### Tracking and Objective/Quantitative Measure

Tracking and objective/quantitative measure: it is possible to record a high quantity of data and use them to create some indexes of performance in order to measure in a quantitative and objective way the improvement of the performances observable in the course of possible rehabilitative process.

### Transferring of the Training in Activity of Daily Living (ADL)

Transferring of the training in activity of daily living (ADL): many studies suggested the potential offered by ecological tasks to transfer the results of re-learning of cognitive and motor abilities that were damaged in ADL. Positive impact of ecological tasks on ADL is documented by many studies (Laver et al., 2015; Chiang et al., 2017).

A previous pilot study investigating the correlation between Neglect App test and classic test in order to understand the usability and ecologicity of our app. Results showed that the cancelation tests of Neglect App were equally effective to the traditional tests in the screening of symptoms between patients with and without neglect. Moreover, the Neglect App Card Dealing task was more sensitive in detecting neglect symptoms than traditional functional task (Pallavicini et al., 2015).

Neglect App contains a series of trials for neglect evaluation through classic tests and virtually interactive environments with the double advantage of automating and making more ecological the evaluation of neglect patients, who thus show a difficulty and/or incapacity to explore, pay attention, perceive, and act in the space region opposite to the area of the brain lesion. Thanks to Neglect App, it is possible to evaluate the explorative behavior of the patient in a fast and simple way, inside ecological environments and receiving all the data, from the performed sessions, included in a database. Neglect App can be downloaded for free at: https://itunes.apple.com/it/app/neglect-app/id788480837?mt=8.

## Results and Discussion

Evaluation is composed of nine exercises divided in two groups: ecological tasks and barrage. The first group comprises ecological tasks, some of which inspired by the ecological battery by Zoccolotti et al., 1994), some others created by starting from real life situations and tasks used clinically but lacking standardization. The app always provides all the score dividing the results in left, right, center, and total areas. Moreover a screenshot with the results is always recorded and generated in the report.

### Ecological Tasks

#### Serve Tea

**FIGURE 1:**
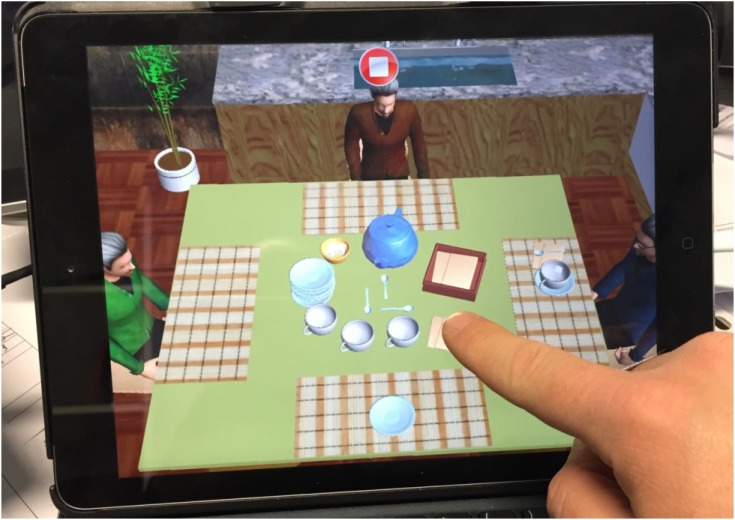
Laying the table tapping on the iPad using the Neglect App.

The patient is required to distribute tea to himself and people sitting at the table with him using objects placed in the center of the table (Figure 1).

The task is commonly used by clinicians in real settings, however, the experience has been replicated in the tablet to be more usable, keeping its own ecological validity.

The patients can be used their finger to drag and drop the single objects for taking the task as requested by the App.

The App already contains the instructions that have to be followed, so the clinicians have just to give the tablet to the patient observing the correct use while executing the task.

Clinicians are not required to take note of the performed actions since the App is able to record every significant action consequently calculating the standard scores that can be used and integrated in a clinical protocol.

**FIGURE 2:**
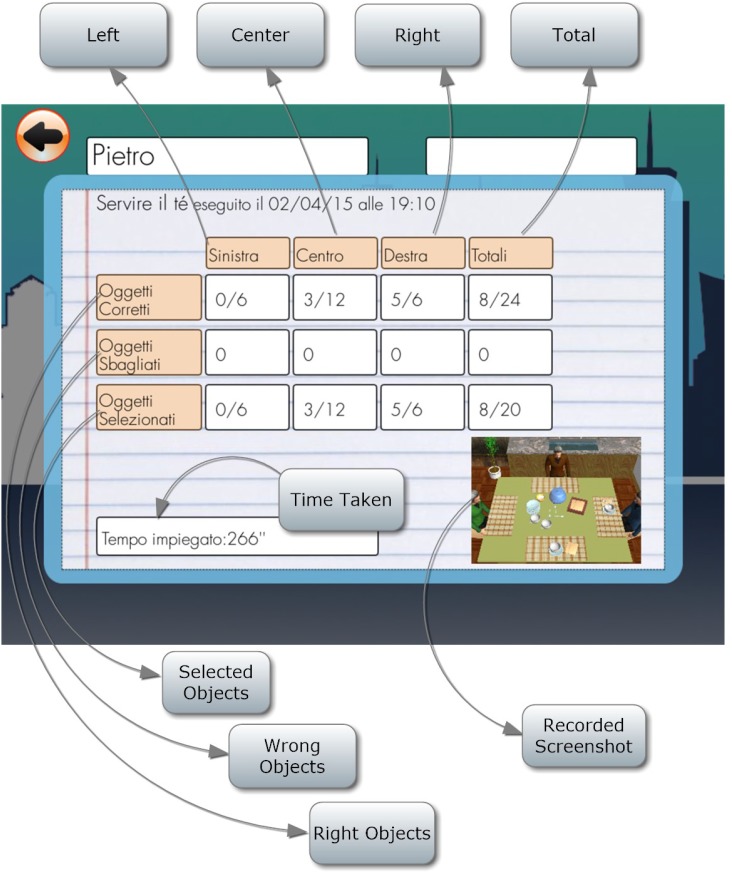
Scores report for the exercise “Distribute the tea.”

The score is assigned on the basis of proper and wrong objects placed to the right, in the center and to the left. Time employed and unconsidered objects are also signaled (Figure 2).

In any time, clinicians are able to access to the patients’ score directly from the App, visualizing each score in each task assigned at any time.

#### Card Dealing

**FIGURE 3:**
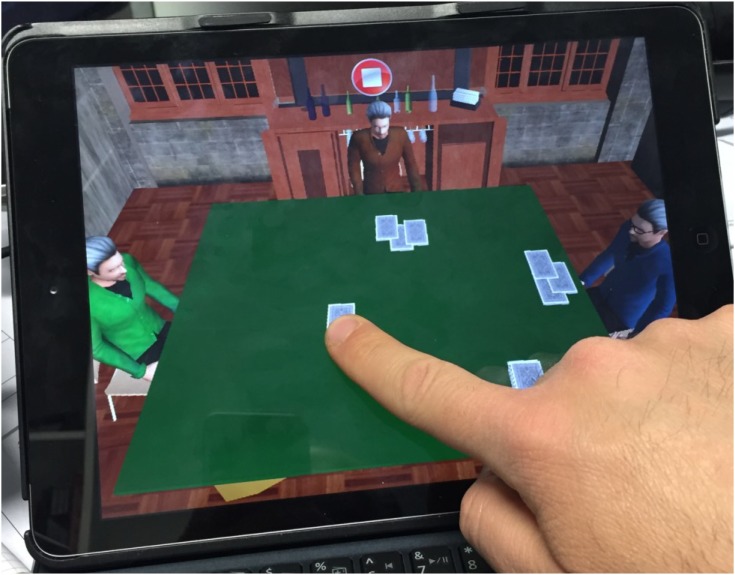
Distributing cards tapping on the iPad using the Neglect App.

The second exercise requires the patient to hand out playing cards to himself and people sitting at the table with him (Figure 3). The score is assigned on the basis of correctly given cards, omitted cards, wrong cards (i.e., those in excess) to the right, to the left and in the middle and the time employed to compete the exercise.

#### Controlling an Orders List

**FIGURE 4:**
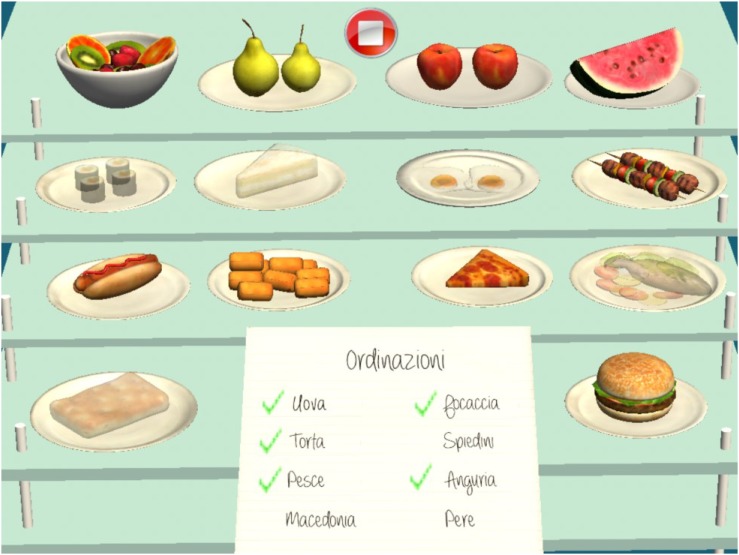
Controlling an orders list task.

In this task, the patient is required to check an orders list to verify if the dishes noted herein are on the shelves; if they are, he/she will have to select the dish on the shelf and the note on the list (Figure 4).

**FIGURE 5:**
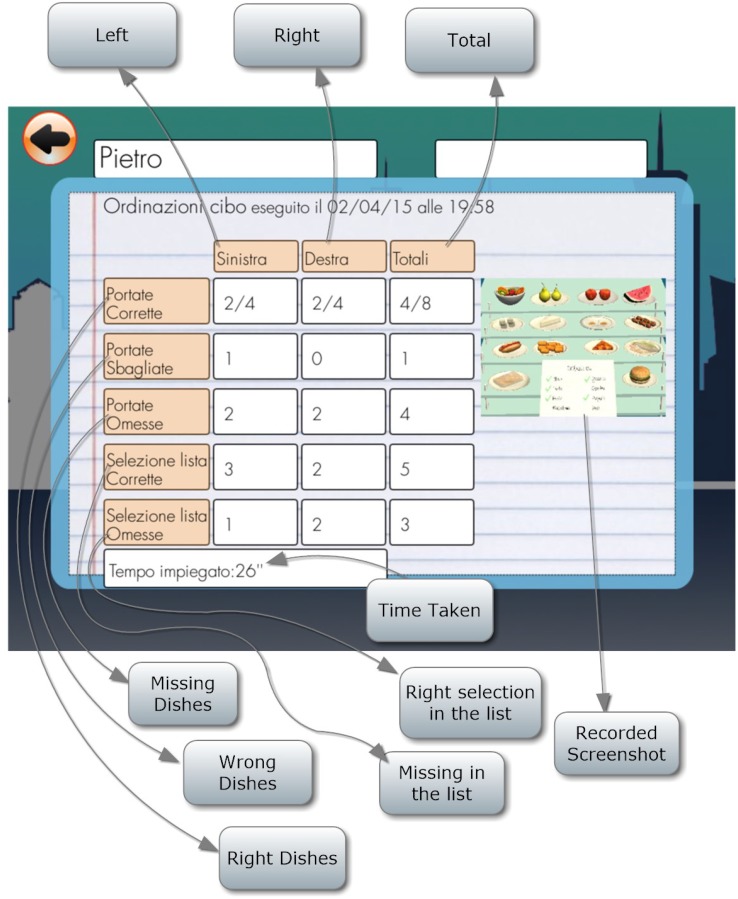
Controlling an orders list score.

Score is assigned on the basis of: the dishes selected correctly; those selected wrongly; the correct dishes omitted; the correct selections and omissions on the list; and the time taken (Figure 5).

#### Exploration

**FIGURE 6:**
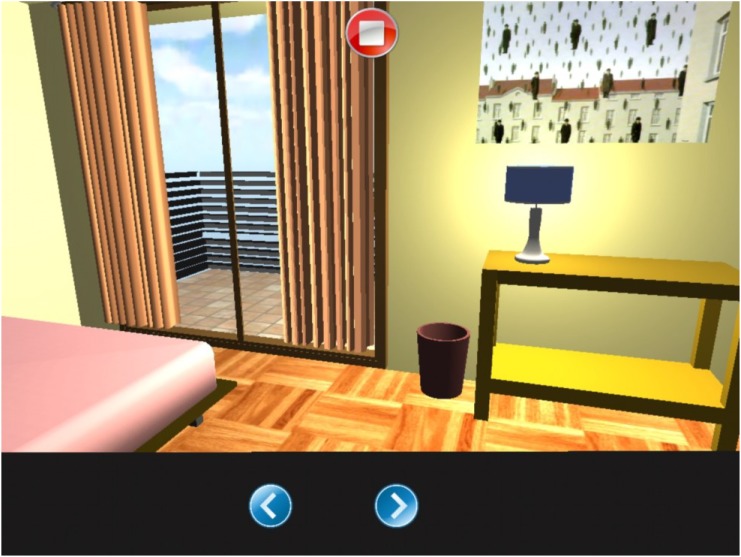
Exploration task.

Within this environment the patient finds him/herself in a room in which he/she can move freely to left or right describing all the objects that are in the room and touching them accordingly (Figure 6). The app calculates automatically, as the patient moves, if the selected object was on the right or the left. The report indicates selected objects on the left, the ones on the right, repetitions, time employed, and omitted elements.

#### Apples Pursuit

**FIGURE 7:**
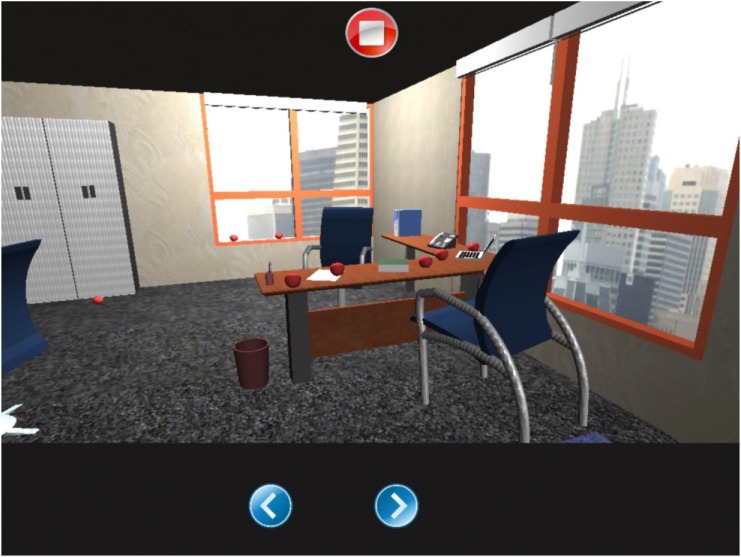
Apples pursuit task.

Within this environment the patient finds him/herself in an office in which he/she can move freely to left or right to identifying and touching all the apples inside (Figure 7). The app calculates automatically, as the patient moves, if the selected apple was on the right or the left. The report indicates selected apples on the left, the ones on the right, repetitions, time employed, and omitted apples.

### Barrage Tasks

Barrage tests take the cue from classical cancelation tasks commonly used clinically (Zoccolotti et al., 1994) and comprehend four exercises, described below.

#### Simple Barrage

**FIGURE 8:**
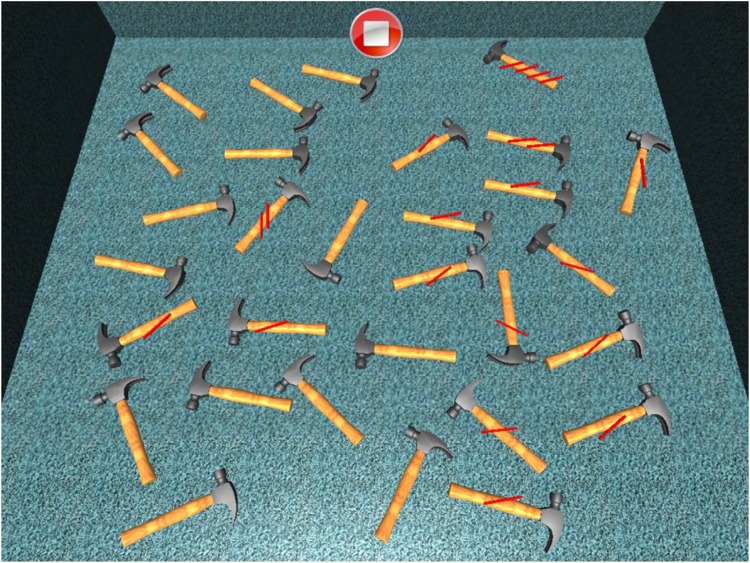
Simple barrage task.

**FIGURE 9:**
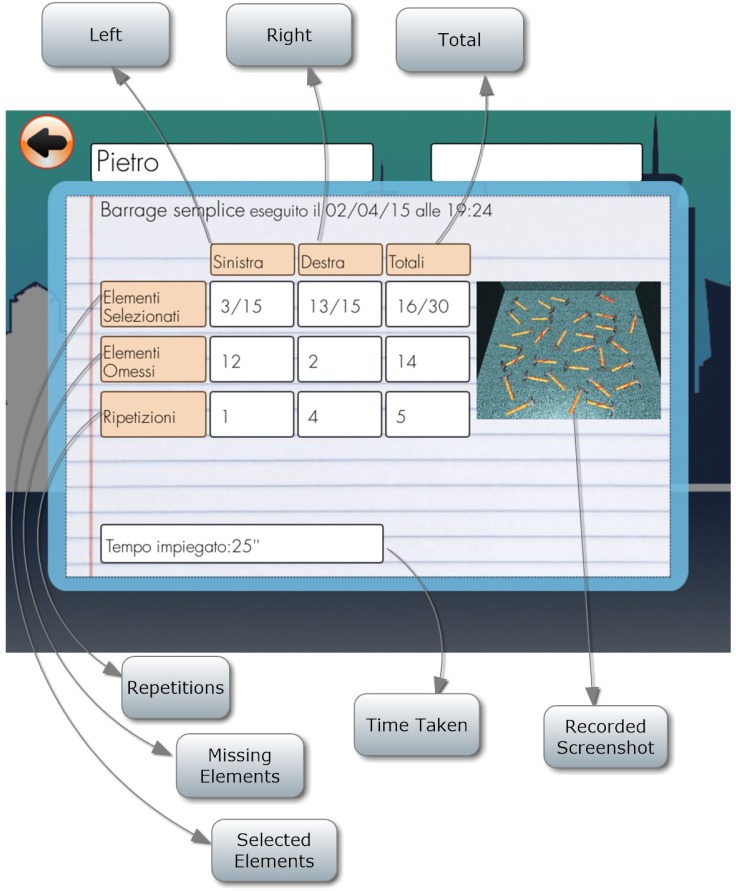
Simple barrage score.

Patient is required to select all objects (hammers) in the room. There are no distractors (Figure 8). The number of selected objects, repetitions, objects omitted on the left and on the right and time employed are considered (Figure 9).

#### Simple Barrage With Distractors

**FIGURE 10:**
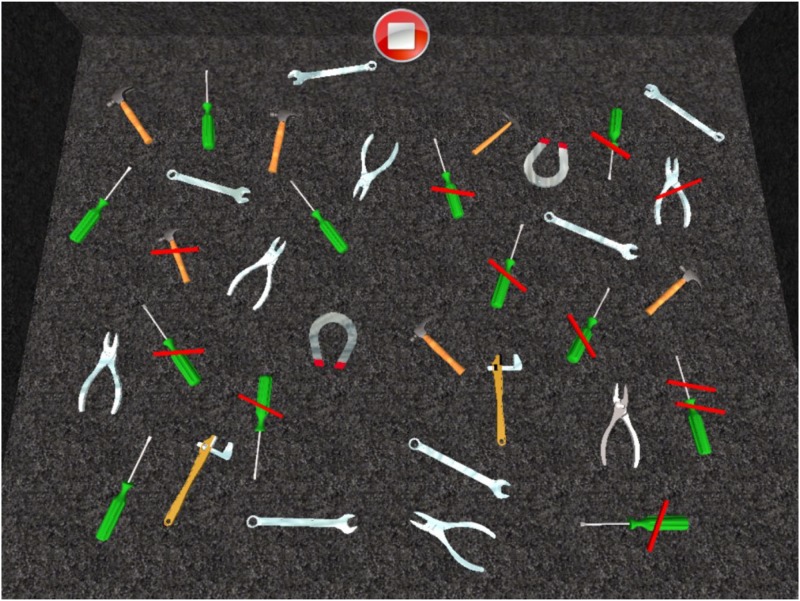
Simple barrage with distractors task.

**FIGURE 11:**
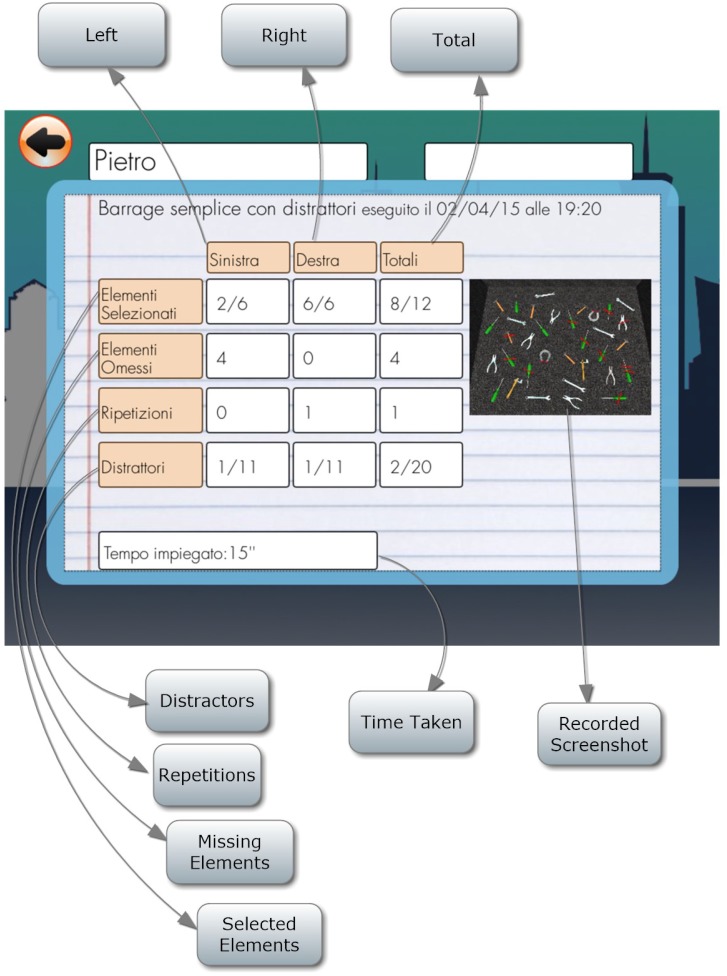
Simple barrage with distractors score.

Patient is required to select all target objects (screwdrivers) in the room, which are mixed with distractors (Figure 10). The number of selected target objects, repetitions, target objects omitted and the distractors selected on the left and on the right and time employed are considered (Figure 11).

#### Dynamic Barrage

**FIGURE 12:**
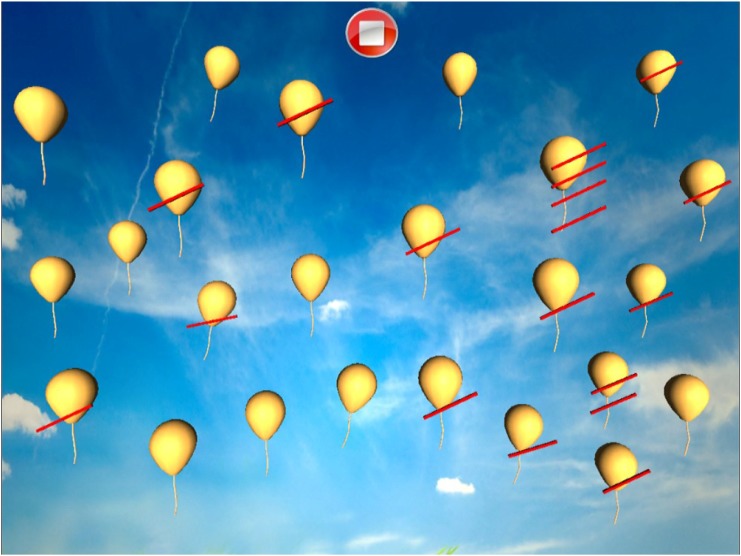
Dynamic barrage task (the balloons are in a continuous movement).

**FIGURE 13:**
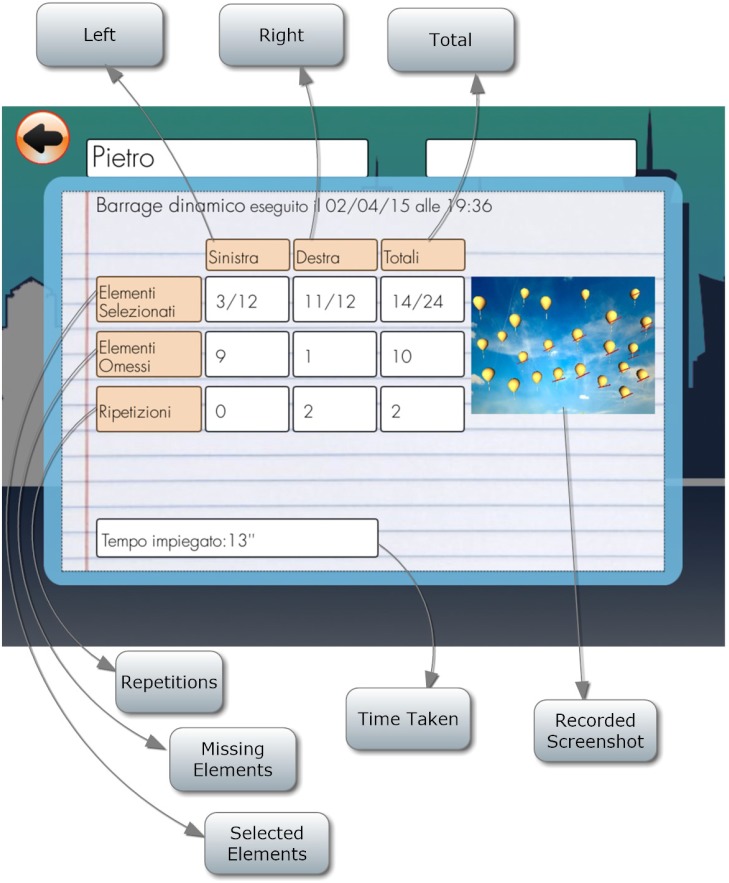
Dynamic barrage score.

Patient is required to select all objects (balloons) in the sky. There are no distractors. The peculiarity here is that the objects are moving (Figure 12). The number of selected objects, repetitions, objects omitted on the left and on the right and time employed are considered (Figure 13).

#### Dynamic Barrage With Distractors

**FIGURE 14:**
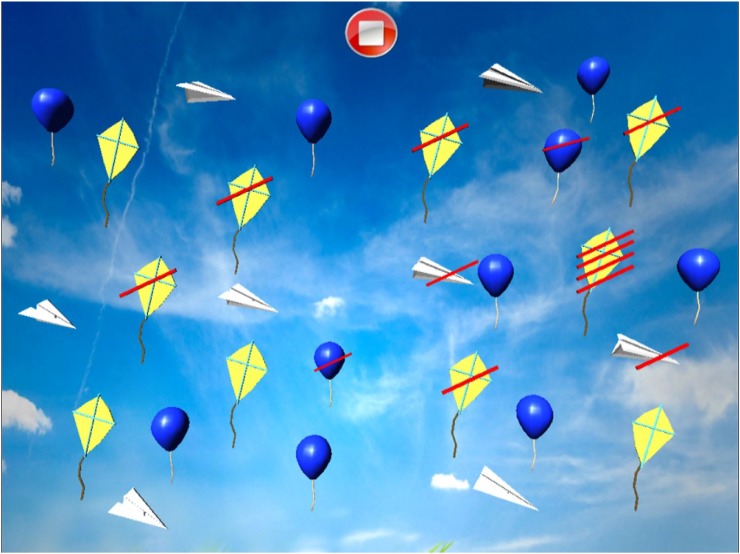
Dynamic barrage with distractors task (all the elements are in a continuous movement).

**FIGURE 15:**
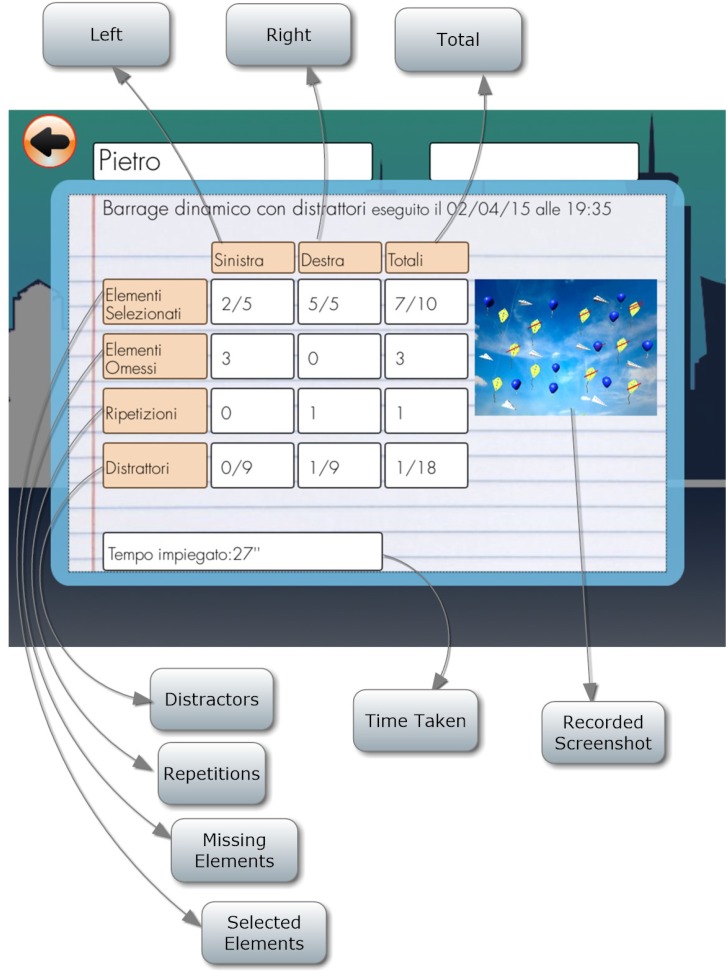
Dynamic barrage with distractors score.

Patient is required to select all target objects (kites) in the room, which are mixed with distractors (Figure 14). The number of selected target objects, repetitions, target objects omitted and the distractors selected on the left and on the right and time employed are considered (Figure 15).

A qualitative analysis of the barrage tasks may give information about dysexecutive behaviors because it is possible to select multiple times every single item and the target in the simple version of both barrage tasks (simple and dynamic) is in the environment of the barrage with distractions tasks.

### Data Management

**FIGURE 16:**
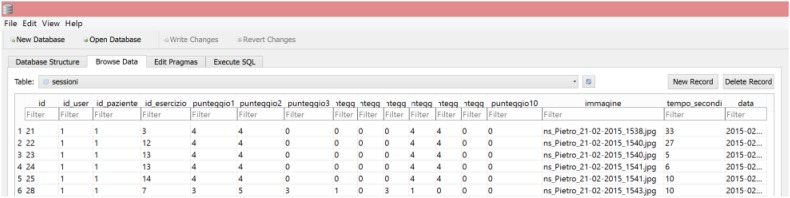
Database management to brows and analyze the data collected.

All data can be downloaded in a unique file by connecting the iPad to a Computer or a Mac equipped with iTunes software. Once downloaded, the file can be easily read with a client software able to interact with SQL Databases (Figure 16). All data, including images, are exportable to be computed for the statistical analysis.

## Conclusion

Neglect may influence the behaviors of the patient in everyday life activity: they can constantly hit the objects placed on his left, not paying attention to the left side of the road when he crosses. In severe cases he can ignore the food in the left half of the plate. So, it has a sufficiently serious framework that allows the patient to cope independently.

The functions such as memory, speech, or attention in neuropsychological research were traditionally assessed through program of standardized tests, which have clear psychometric advantages, but often measure behaviors that are very different from those of everyday life (Chaytor and Schmitter-Edgecombe, 2003).

**FIGURE 17:**
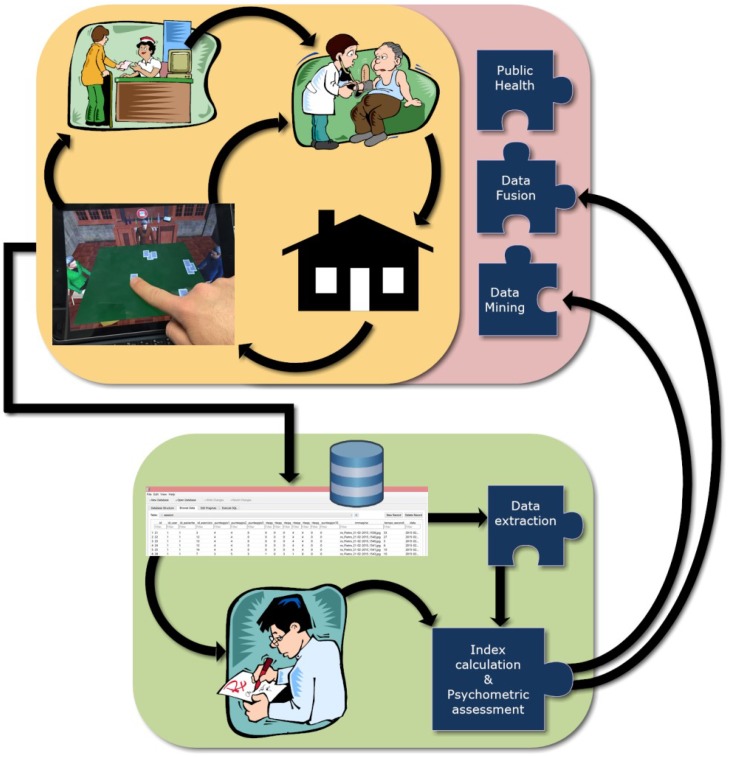
The Neglect App cycle. From the patients into the clinic to the continuous assessment at the patient’s home.

In recent years, there has been a growing interest in the development of tools that allows ecological and functional assessment above all by using mobile device (Villani et al., 2012a, 2013; Carbonaro et al., 2014; Pedroli et al., 2015b). The results of a meta-analytic review of Neguţ et al. (2016) support the sensitivity of virtual reality tools in detecting cognitive deficit. One of the areas where emerges this need is the assessment of neglect. We decided to diffuse the application in the Italian market with a future intention to extend worldwide a possible English version. The Neglect App temporal cycle concern from the moment of the patients into the Clinique to the continuous assessment at the patient’s home and back to the Clinique in a closed loop for the continuous assessment (Figure 17).

Assessment by using a mobile tool and virtual environments might represent a great challenge for very sophisticated methods able to assess in a way before unthinkable and sometimes impossible in real settings. In particular, navigation tasks allow the system to identifying if an object in the space is located in left or right side when selected. On the other hand, in real settings to do this navigation task is too expensive, requiring eye-tracking glasses. Moreover a computational approach can be easily used to provide more feedback to the patients and to model behaviors (Cipresso, 2015; Cipresso et al., 2015).

One of the limitations of the App is the screen dimension, that does not provide any direct advantage compared to paper and pencil test. Actually, this limitation has been recently overcome by the iPad Pro 12,9′′ that can be effectively used with our App, being totally compatible. Another limitation is the lack of normative data available for a quantitative analysis of the results; only a qualitative analysis is recommended. A future study could be able to fill this gap.

At the moment we have not implement some additional information and indexes that could help the clinicians to better understand the characteristics of their explorative behaviors in order to program a more personalized rehabilitations. In particular, it could be interesting to report the starting point and the path of the exploration made by patients or some other indexes like the ones reported in the Chung et al.’s (2016) article.

Additionally, to create some tasks for rehabilitations could make our application completer and more interesting. Provide some tasks for make exercises in a virtual environment could help patients and clinicians to improve clinical practice.

The future development will have directed to fill these limitations with the addition of some specific tasks both for assessment and rehabilitation. A manipulation of the cognitive complexity of the barrage tasks according to the criteria proposed by Ricci et al. (2016) and Sarri et al. (2009) could help to have a more precise assessment process. To aim this scope the introduction of a 3D version of line bisection task are also consider because some patients may show neglect symptoms in this kind of task and not in the barrage one.

Also, a new version developed to take advantage of the immersive technology could be designed in order to reach a higher degree of ecologicity.

After all these modifications a validation study will be necessary in order to prove the validity of our system. Also, a clinical trial for the rehabilitation session could be done in order to prove the usefulness of a computerizing protocol. Both, convergent and discriminant validity, need to be verified comparing current tools accordingly. At this purpose can be used current neuropsychological battery and specific test, such as barrage test, front assessment battery, real task (e.g., lay the table in real context), and so on.

We are so providing the scientific and clinical communities a free advanced tool able to be a practical and flexible way for the assessment directly in the patients’ place but also a brand-new way for the assessment of Neglect.

### Availability and Requirements

• **Project name:** Neglect App.
• **Project home page:** https://itunes.apple.com/it/app/neglect-app/id788480837?mt=8.
• **Operating system(s):** iOS Platform (at least iOS 6.0 is required).
• **Programming language:** No programming language is required for using the App. The Neglect App has been developed by using Unity.
• **Other requirements:** the App works also on iPhone device but iPad device is suggested for the best use and visualization.
• **License:** Available for free.
• **Any restrictions to use by non-academics:** No restrictions.

## Author Contributions

PC and EP wrote the manuscript. PC, EP, SS, MS, CT, and DC collected the literature materials. GR supervised the study. PC, EP, SS, FP, and GR conceived the idea of the study, established the software requirements, and supervised the technological, clinical, and scientific aspects. All authors read and approved the final manuscript.

## Conflict of Interest Statement

The authors declare that the research was conducted in the absence of any commercial or financial relationships that could be construed as a potential conflict of interest.
